# The Preventive Effect of Biochanin A on Bone Loss in Ovariectomized Rats: Involvement in Regulation of Growth and Activity of Osteoblasts and Osteoclasts

**DOI:** 10.1155/2013/594857

**Published:** 2013-02-21

**Authors:** Shu-Jem Su, Yao-Tsung Yeh, Huey-Wen Shyu

**Affiliations:** Department of Medical Laboratory Science and Biotechnology, School of Medicine and Health Sciences, Fooyin University, No. 151, Chinhsueh Road, Ta-liao, Kaohsiung 83101, Taiwan

## Abstract

Biochanin A (BCA) is a major isoflavone abundant in red clover (*Trifolium pretense*). The protective effect of BCA on bone loss in an ovariectomized (OVX) animal model has never been clarified. The objective of this study was to investigate the biological effects of BCA on bone loss in OVX rats *in vivo* and on the development of osteoblasts and osteoclasts *in vitro*. Ovariectomy resulted in a marked increase in body weight and a decrease in femoral bone mineral density and trabecular bone volume that was prevented by BCA or 17**β**-estradiol (E2) treatment. However, an increase in uterine weight was observed in E2-treated OVX rats, but not in response to BCA treatment. Treatment with BCA increased the mRNA expression of osterix, collagen type I, alkaline phosphatase (ALP), and osteocalcin and decreased the mRNA expression of tartrate-resistant acid phosphatase (TRAP) and the receptor activator of nuclear factor-**κ**B ligand (RANKL)/osteoprotegerin (OPG) ratio in the femur of OVX rats. Treatment with BCA or E2 prevented the OVX-induced increase in urinary deoxypyridinoline (DPD) and serum tumor necrosis factor **α** (TNF-**α**) and interleukin-1**β** (IL-1**β**). *In vitro*, BCA induced preosteoblasts to differentiate into osteoblasts and increased osteoblast mineralization. BCA inhibited preosteoclasts and osteoclast proliferation and decreased osteoclast bone resorption. These findings suggest that BCA treatment can effectively prevent the OVX-induced increase in bone loss and bone turnover possibly by increasing osteoblastic activities and decreasing osteoclastic activities.

## 1. Introduction

Osteoporosis is the most common metabolic bone disease in women. A menopausal decline in estrogen contributes significantly to an increased rate of bone remodeling and leads to an imbalance between bone resorption and formation, eventually causing bone loss and osteoporosis [1, 2]. High bone turnover, with increased bone resorption, can compromise bone strength, leading to a thinning of the bone structure, resulting in abnormal bone microarchitecture and reduced bone mineralization. This, in turn, leads to a greater propensity to fracture.

Phytoestrogens are natural plant-derived products that have structural and functional similarities to estradiol. They are considered to be a safe alternative to hormone replacement therapy (HRT) [3] and are used in phytomedicine to treat menopausal symptoms and osteoporosis. Our previous study had demonstrated that soy isoflavone extract in combination with vitamin D3 was able to decrease bone loss during estrogen deficiency in an animal model [4], implicating that isoflavone might be an alternative to HRT. Biochanin A (BCA), a major isoflavone found in red clover (*Trifolium pretense*) and many other legumes, is commercially available as a nutraceutical and is known to exert positive health effects and may be useful in the maintenance of bone health [5]. We suggested that BCA may have benefits to osteoporosis resulted from estrogen decline.

Red clover has a higher content of BCA (5,7-dihydroxy-4′-methoxyisoflavone) and formononetin (7-hydroxy-4′-methoxyisoflavone) and a lower content of genistein (5,7,4′-trihydroxyisoflavone) and daidzein (4′,7-dihydroxyisoflavone) than soy [6]. Previous studies have shown that clover-derived isoflavone supplements provide potential benefits to bone. Red clover-derived isoflavone supplements reduce the loss of lumbar spine bone mineral content (BMC) and bone mineral density (BMD) in women [7, 8] and improve OVX-induced osteoporosis [9]. The mechanism by which these red clover phytoestrogenic isoflavones are effective at reducing bone loss induced by ovariectomy is thought to be by reducing bone turnover via inhibition of bone resorption [10]. Limited bone metabolism data have been reported for BCA. *In vitro* studies showed that BCA stimulated differentiation of osteoblastic MC3T3-E1 cell line [11] and modulated lipid metabolism [12]. However, the biological roles of BCA in the enhancement of bone formation *in vivo* and the attenuation of bone resorption *in vivo* and *in vitro* are largely unknown. 

Although BCA is converted into the demethylated metabolite genistein, the biological effects of BCA observed *in vivo* are not identical to those of genistein [13]. BCA is thought to exert its beneficial effects predominantly through the estrogen receptor beta (ER*β*) (found in bone and blood vessels) [14, 15]; the affinity of BCA for ER*β* is greater than that for ER*α* (found in breast and uterus). Previous studies have shown that BCA has vasculoprotective effects without uterotrophic activity [14, 16]; thus, administration of ER*β*-selective agents might be alternative treatments to reduce the risk of cardiovascular disease and bone loss in postmenopausal women. 

The aim of the present study was to evaluate the possible beneficial effect of BCA on bone loss in the ovariectomized rat model of osteoporosis. This model is the most commonly used model for the study of human postmenopausal osteoporosis [17]. In addition, plasma BCA concentrations lower than or equal to 10^−6^ M are attainable with a daily oral intake of 5–50 mg per kg of body weight in rats [18]. Indeed, the maximum plasma concentration of any isoflavone rarely exceeds 10^−6^ M following dietary intake [19]. Thus, we further examined the effectiveness and underlying molecular mechanism of BCA (at a physiologically relevant concentration of 10^−6^ M or lower) in bone health by using two major osteoporosis-related primary bone cells, osteoblasts, and osteoclasts. 

## 2. Materials and Methods

### 2.1. Animals and Treatments

The animals and treatment methods used were described in a previous study [4]. Virgin female Sprague-Dawley (SD) rats aged 3 months (280–300 g) were sham-operated (*n* = 10) or bilaterally ovariectomized (*n* = 30) as an experimental animal model of estrogen depletion-induced bone loss. The OVX rats were randomly assigned to one of three treatment groups: untreated, treated for 14 weeks with E2, and treated for 14 weeks with BCA (Sigma-Aldrich, St. Louis, MO, USA). The sham-operated, OVX control and E2-treated rats received a control diet. The E2-treated rats received intraperitoneal injection of E2 (23 *μ*g/kg body weight per day) (Sigma-Aldrich) on 3 consecutive days per week; this dose was chosen to replace approximately 90% of the estrogen loss observed after ovariectomy. The BCA-treated group received daily oral administration of BCA (25 mg/kg body weight per day).

All rats were housed individually in metal cages. Animals were maintained at 23°C, on a 12 h light:dark cycle with standard rat food pellets. Water was provided *ad libitum*. This procedure was approved by the Kaohsiung Medical University Animal Care and Use Committee.

The rats were sacrificed by an overdose of CO_2_ at the end of the study. Blood was collected in a heparinized syringe from the abdominal aorta, transferred to a glass tube for 1 h, and then centrifuged at 3000 g for 10 min at 4°C to separate the serum. The left femur was removed for histological study and for extraction of mRNA and protein. All samples were stored at −80°C until further analyzed.

### 2.2. Body Weight, Uterine Weight, Bone Mineral Density, Bone Mineral Content and Bone Volume

Body weight was measured on a weekly basis to monitor health and measure weight gain. At the end of the study, the rats were anesthetized and scanned under a dual energy X-ray absorptiometer (Norland XR-36; Norland, Fort Atkinson, WI, USA). The left femur was examined for BMD and BMC, and PMOD image analysis software (PMOD Technologies, Zurich, Switzerland) was used to calculate bone volume by selecting the same position of the left femur in each rat. The percentage of the trabecular bone volume in relation to the total tissue volume examined (%BV/TV) was measured. Uterine weights were determined after removal of the uterus. 

### 2.3. Serum and Urinary Biochemical Markers

Serum calcium and inorganic phosphorous concentrations were measured using an autoanalyzer (7070; Hitachi, Tokyo, Japan). Urine samples were individually collected from rats housed in metabolic cages during the 24 hours preceding sacrifice. Urinary deoxypyridinoline (DPD) levels were measured by ELISA (DPD EIA kit, Metra Biosystems, US). Commercial kits were used to analyze serum concentrations of TNF-*α* and IL-1*β* (R&D, Minneapolis, MN, USA).

### 2.4. Reverse Transcriptase-Polymerase Chain Reaction

Total RNA was extracted from the left femur using REzol reagent (Protech, Taiwan). Reverse transcriptase-polymerase chain reaction (RT-PCR) was performed as described previously [20]. To synthesize complementary DNA (cDNA), 2 *μ*g of RNA was resuspended in 12.5 *μ*L of diethylpyrocarbonatetreated water, 1 *μ*L of oligo(dT) primer was added, and the mixture was annealed for 5 min at 70°C. The sample was then cooled to 4°C for 2 min before addition of 4 *μ*L of 5x reaction buffer (50 mM Tris-HCl, 75 mM KCl, 3 mM MgCl_2_, pH 8.3), 0.5 *μ*L of RNase inhibitor, 1 *μ*L of 10 mM dNTP, and 1 *μ*L of Maloney's murine leukemia virus reverse transcriptase (Promega, Lyon, France). The reaction mixture was heated for 60 min at 37°C to synthesize the cDNA, and the reaction was stopped by denaturing the enzyme at 94°C for 5 min. cDNA was amplified by PCR to generate the genes listed as follows: Osterix (NM_001173467): forward: 5′-ggaggcaactggctag-3′, reverse: 5′-gctgcccactatttccc-3′ (229 bp); ALP (NM_000478): forward: 5′-ctctccgagatggtgg-3′, reverse: 5′-tggagacattctctcgtt-3′ (373 bp); collagen type I (NM_000088): forward: 5′-ggctatgatgagaaatcaa-3′, reverse: 5′-atccaaaccactgaaacc-3′ (266 bp); Osteocalcin (NM_199173): forward: 5′-tgcagagtccagcaaag-3′, reverse: 5′-gctccaggggatccg-3′ (125 bp); OPG (NM_002546): forward: 5′-ctgtgtgaggaggcatt-3′, reverse: 5′-agctgtgttgccgtttta-3′ (136 bp); RANKL (NM_033012): forward: 5′-agcatcaaaatccca agtt-3′, reverse: 5′-aactttaaaagccccaaag-3′ (204 bp); and TRAP (NM_001111034): forward: 5′-gccatttttatgctggac-3′, reverse: 5′-cttgaccaggcagtgg-3′ (216 bp). GAPDH (NM_001256799): forward: 5′-atggtttacatgttcc aata-3′, reverse: 5′-ctcgctcctggaaga-3′ (115 bp) was amplified as a housekeeping gene. PCR amplification was performed for 35 cycles at 94°C for 1 min, 52°C for 1 min, and 72°C for 1 min, followed by 7 min at 72°C. The amplified PCR products were separated by gel electrophoresis in 2% agarose gel and visualized with ethidium bromide, and the intensity of each band was calculated by densitometry analysis and the results were expressed as a percentage of the density of the corresponding GAPDH band.

### 2.5. Bone-Cell Culture and Proliferation

To analyze the direct effects of BCA or E2 on the differentiation and function of osteoblasts and osteoclasts, rat primary osteoblast and osteoclast progenitors were isolated and cultured. The rat calvarial osteoblast culture system was as described by Udagawa et al. [21], and the osteoclast culture system using the osteoclastogenic agents 1*α*,25-dihydroxyvitamin D_3_ (10 nmol/L), dexamethasone (10 nmol/L), and macrophage-colony stimulating factor (10 ng/mL) was as described by Zou and Bar-Shavit [22]. Preosteoblasts or mature osteoblasts were seeded onto 24-well plates (5 × 10^3^ cells/well) in minimum essential medium containing 10% fetal calf serum and were allowed to attach for 24 h. Incubation was continued for another 48 h in the presence or absence of physiological concentrations of 10^−8^–10^−6^ M BCA. Preosteoclasts or mature osteoclasts were treated identically with the exception of inclusion of osteoclastogenic agents in the seeding medium. Crystal violet uptake by the cells was used to evaluate cell number.

### 2.6. Enzyme Histochemistry

Preosteoclasts or preosteoblasts were seeded onto 24-well plates (1 × 10^3^/well) in minimum essential medium containing 10% fetal calf serum and were allowed to attach for 24 h. Incubation was continued for 7 days with or without 10^−8^–10^−6^ M BCA. Fresh medium was supplied at 3-day intervals. Multinucleated cells were fixed in formalin/acetone/citric acid and checked for the presence of the osteoclast marker enzyme, TRAP, or the osteoblast marker, ALP, by assaying enzyme activity using commercially available kits (Sigma-Aldrich). Positive cells were stained brown or red. TRAP^+^-multinucleated cells and ALP^+^ osteoblasts were counted using a semiautomatic image-analyzing program.

### 2.7. Cellular ALP and TRAP Activity

Osteoclasts or osteoblasts (5 × 10^4^/well) were seeded onto a 24-well plate in minimum essential medium containing 10% fetal calf serum or osteoclastogenic agents. After incubation for 24 h, fresh medium with or without the test agents at 10 mmol/L was supplied until the cells reached confluence. The TRAP activity of osteoclasts or the ALP activity of osteoblasts was measured using a p-nitrophenyl phosphate kit (Sigma-Aldrich).

### 2.8. Cell Migration Assay for Osteoblasts

Cell migration was determined following the method of Xu et al. [23]. Osteoblasts were detached and 5 × 10^5^ cells were seeded onto Corning 24-well tissue culture plates. Incubation was continued for 48 h in fresh medium with or without 10^−6^ M BCA. The cell migration area was counted using a semiautomatic image-analyzing program. There were 24 wells per treatment group. All experimental protocols were repeated at least three times.

### 2.9. Mineralization Assay

The von Kossa mineralization assay was followed as described by Bellows et al. [24]. The phosphorous calcium deposition area was measured using a semiautomatic image-analyzing program (Mac Scope, Mitani, Fukui, Japan).

### 2.10. Bone Resorption Assay

After treatment, the osteoclasts were lysed with distilled water and the slides were stained with 5% aqueous AgNO_3_, exposed to ultraviolet light for 60 min at room temperature, and then incubated in 2.5% sodium thiosulfate for 5 min. The area of the resorption pits was measured using a Zeiss Morphomat 10 (Carl Zeiss, La Pecq, France) for 100–150 pits selected at random on each slide. The pit area was counted using a semiautomatic image-analyzing program (Mac Scope). Each experiment was performed at least three times. 

### 2.11. Statistics

The results of the *in vivo* and *in vitro* data are presented as the mean ± standard deviation (SD). Differences among the groups (Sham, OVX, OVX+E2, and OVX+BCA) were analyzed statistically using one-way analysis of variance (ANOVA), followed by Fisher's test. A *P* value of <0.05 was considered statistically significant.

## 3. Results

### 3.1. Body Weight and Uterine Weight in OVX Rats

At 14 weeks after bilateral ovariectomy, serum estrogen levels had dramatically decreased from 56.3 ± 5.6 pg/mL to 3.0 ± 2.5 pg/mL. In accordance with reports that estrogen modulates lipid metabolism [25, 26], the body weight was markedly increased by 63.8 ± 7.2% compared to an increase of 33.6 ± 5.0% in the sham group. Treatment with BCA or E2 significantly decreased the body weight of OVX rats (*P* < 0.05) (Table 1). In addition, uterine weight was significantly reduced in OVX rats (*P* < 0.05). Treatment of OVX rats with E2 significantly increased uterine weight compared to OVX rats (*P* < 0.05), but uterine weight was unchanged in BCA-treated OVX rats.

### 3.2. Femur BMD, BMC, and BV/TV in OVX Rats

The left femur BMD and BMC were measured by dual energy X-ray absorptiometry. The results listed in Table 2 show that BMD of the OVX group was markedly reduced by 14.5% in comparison to that in the sham group (*P* < 0.05). Treatment with BCA or E2 for 14 weeks maintained BMD levels similar to those of the sham group (*P* < 0.05). Bone mineral content of the OVX group was significantly lower (*P* < 0.05) than that of the sham group, but treatment with BCA or E2 effectively increased BMC in the OVX group (*P* < 0.05). Computed tomography of the distal femur showed that %BV/TV was markedly decreased by ovariectomy (Table 2), suggesting the induction of osteopenia. Treatment with BCA or E2 resulted in a significant increase in %BV/TV compared with the OVX control (*P* < 0.05).

### 3.3. Serum and Urinary Biochemical Markers in OVX Rats

There was no significant difference in serum calcium or phosphate levels among all groups (Table 3). The bone resorption marker, urinary DPD, was increased in the OVX group and diminished by treatment with BCA or E2 (Table 3). Serum levels of cytokines TNF-*α* and IL-1*β*, which are responsible for enhanced osteoclastogenesis and activation of mature osteoclasts for bone resorption, were significantly increased in OVX rats and diminished by treatment with BCA or E2 (*P* < 0.05) (Table 3).

### 3.4. Osteogenic Marker mRNA Levels in OVX Rats

**Figure 1:**
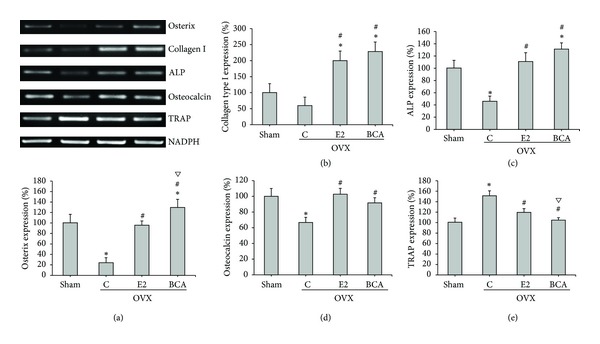
Effects of BCA and E2 on osteoblast and osteoclast marker gene expression. mRNA expression in the distal femur was determined by RT-PCR for (a) osterix, (b) collagen type I, (c) ALP, (d) osteocalcin, or (e) TRAP. Expression was normalized to that of GAPDH and expressed as a percentage of that in the sham group. The bars represent mean ± SD for ten samples. *P < 0.05 compared with the sham group; ^#^ *P* < 0.05 compared with the OVX C group; ^*∇*^ *P* < 0.05 compared with the E2 group. ALP: alkaline phosphatase; TRAP: tartrate-resistant acid phosphatase; C: control; BCA: biochanin A; E2: 17*β*-estradiol; OVX: ovariectomized.

The mRNA levels of osteoblast and osteoclast marker genes in distal femur bone tissue were determined by RT-PCR. Expressions of the osteoblast osteogenic genes osterix, collagen type I, ALP, and osteocalcin were remarkably decreased in the untreated OVX group compared to those in the sham group (Figures 1(a)–1(d)). These reductions were prevented upon treatment with BCA or E2. However, osterix and ALP expression levels in the BCA-treated groups were higher than those in the sham- and E2-treated group (*P* < 0.05). Expression of the osteoclast marker gene, TRAP, was increased in the untreated OVX group and this increase was prevented by E2 or BCA (Figure 1(e)). 

### 3.5. Expression of RANKL/OPG during Bone Turnover in OVX Rats

**Figure 2:**
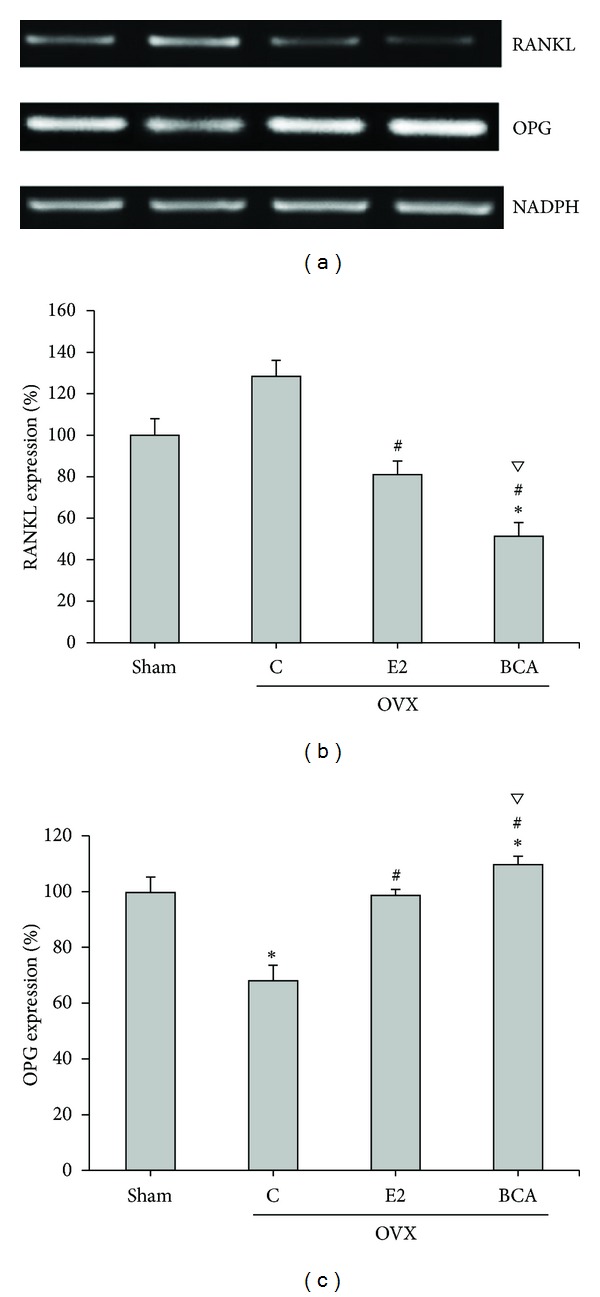
Effects of BCA and E2 on RANKL and OPG mRNA expression. The mRNA levels for RANKL (b) and OPG (c) in the distal femur were determined by RT-PCR. Expression was normalized to that of GAPDH and expressed as a percentage of that in the sham group. The bars represent the mean ± SD for ten samples. *P < 0.05 compared with the sham group; ^#^ *P* < 0.05 compared with the OVX C group; ^*∇*^ *P* < 0.05 compared with the E2 group. RANKL: receptor activator of nuclear factor-*κ*B ligand; OPG: osteoprotegerin; C: control; BCA: biochanin A; E2: 17*β*-estradiol; OVX: ovariectomized.

Studies in normal healthy animals have revealed that OPG and RANKL play important ongoing roles in the maintenance of bone mass and in the regulation of normal bone remodeling [27]. Ovariectomy is associated with increased bone turnover and reduced BMD, volume, and strength. Ovariectomy has been shown to increase RANKL levels and decrease OPG levels in various animal models [28, 29]. The RANKL/OPG ratio is an index of osteoclastogenic stimulation and an increase RANKL/OPG ratio is observed during bone resorption in ovariectomy. Hence, we further investigated expression of RANKL and OPG *in vivo*. RANKL and OPG mRNAs were prepared from rat distal femur bone tissue and were determined by RT-PCR. The RANKL/OPG ratio was remarkably increased in the untreated OVX group compared to that in the sham group, whereas treatment with BCA or E2 significantly decreased the RANKL/OPG ratio (Figure 2). This suggested that both BCA and E2 decreased bone resorption during bone turnover in OVX rats.

### 3.6. Preosteoblast and Osteoblast Proliferation and Differentiation

**Figure 3:**
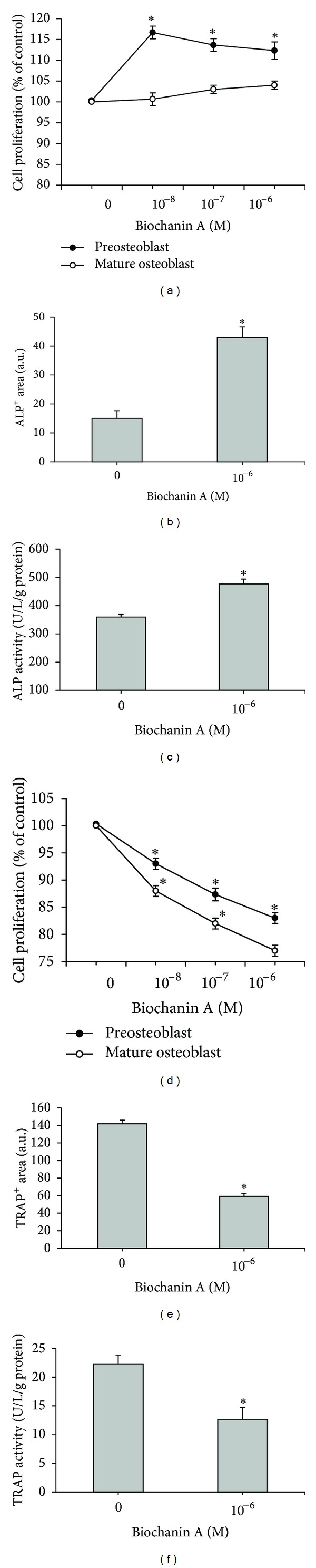
Effects of BCA on (a) the growth of preosteoblasts and mature osteoblasts, (b) preosteoblastic ALP staining, (c) mature osteoblastic ALP^+^-specific activity, (d) the growth of preosteoclasts and mature osteoclasts, (e) preosteoclastic TRAP^+^ staining, and (f) mature osteoclastic TRAP activity. All data are shown as mean ± SD for three separate experiments. *P < 0.05 compared with the control. BCA: biochanin A; ALP: alkaline phosphatase; TRAP: tartrate-resistant acid phosphatase.

After culture, the osteoblast precursor cells started developing into ALP^+^ preosteoblasts and osteoblasts. These cells were incubated with or without various concentrations of BCA or E2 for 2 days to examine the effects of BCA or E2 on cell proliferation, or for 7 days to examine the effects of BCA or E2 on expression of ALP, a marker of osteoblast differentiation. BCA (10^−8^–10^−6^ M) significantly increased preosteoblast cell proliferation (*P* < 0.05) and slightly enhanced osteoblast proliferation, but this was not significant compared to the control (*P* > 0.05) (Figure 3(a)). Expression and activity of ALP were significantly increased after incubation with 10^−6^ M BCA compared to the control (*P* < 0.05) (Figures 3(b) and 3(c)).

### 3.7. Preosteoclast and Osteoclast Proliferation and Differentiation

Osteoclast formation is known to be stimulated by 1*α*,25-dihydroxyvitamin D_3_ [30]. In the present study, we found that osteoclastogenic agents including 10 nmol/L 1*α*,25-dihydroxyvitamin D_3_, 10 nmol/L dexamethasone, and 10 nmol/L M-CSF induced the differentiation of bone marrow cells into TRAP^+^ osteoclastic cells. BCA was added to the osteoclastogenic agent-treated cells, and these cells were cultured for an additional 2 days to examine the effects of BCA on cell proliferation. Cells were cultured for 7 days in order to analyze the effects of BCA on TRAP. The preosteoclastic cell proliferation was dose-dependent and remarkably inhibited in the presence of BCA (10^−8^–10^−6^ M) (*P* < 0.05) (Figure 3(d)). In addition, BCA also inhibited the proliferation of mature osteoclasts in a dose-dependent manner (Figure 3(d)). Notably, TRAP is a marker enzyme for the differentiation and formation of osteoclasts, and TRAP expression and activity were also decreased after incubation for 7 days with 10^−6^ M BCA (*P* < 0.05) (Figures 3(e)-3(f)).

### 3.8. Osteoblast Migration, ALP Activity, and Mineralization

**Figure 4:**
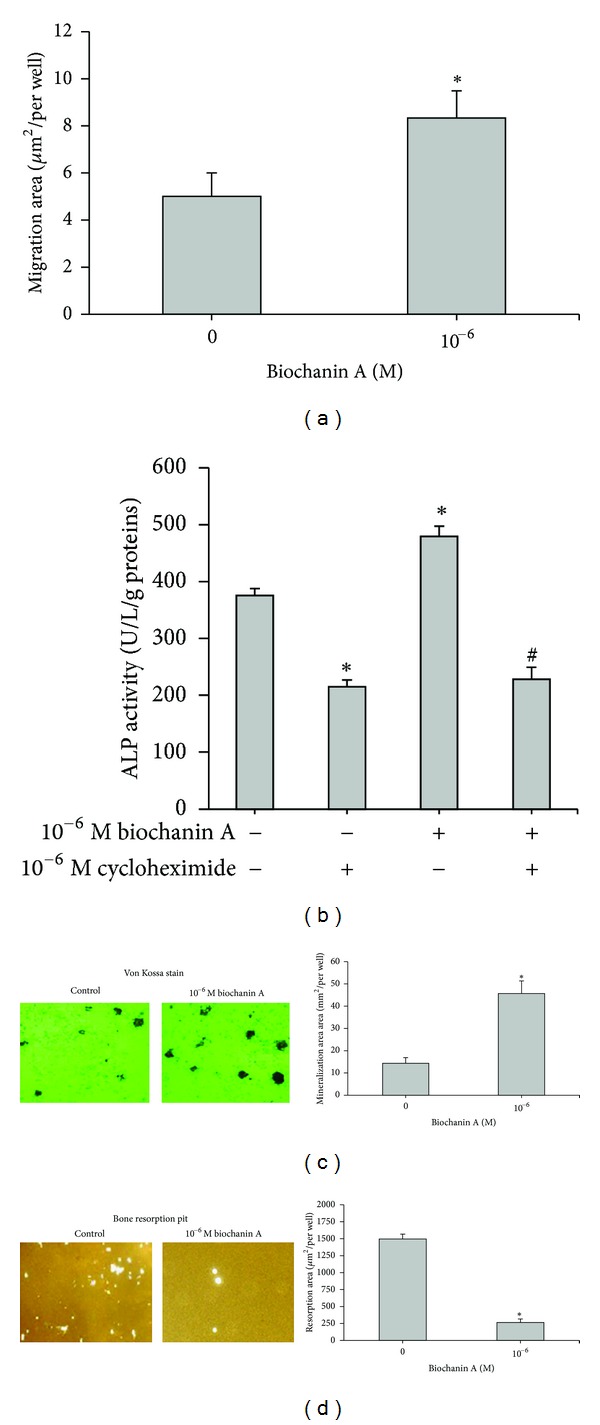
Effects of BCA on the functional activities of osteoblasts and osteoclasts. (a) Osteoblasts were incubated for 2 days with or without 10^−6^ M biochanin A, and then cell migration was evaluated as described in Section 2. (b) Osteoblasts (5 × 10^4^ cells/well) were incubated for 7 days with 10^−6^ M BCA in the presence or absence of cycloheximide and then assayed for ALP activity. (c) Osteoblasts were cultured with or without 10^−6^ M BCA for 7 days, followed by evaluation of von Kossa staining for mineralized nodules. (d) Osteoclasts were cultured with or without 10^−6^ M BCA for 7 days, and the surface of the resorption pit area was determined. (Left) Representative photographs of stained cells. (Right) Summarized data expressed as mean ± SD for three separate experiments. *P < 0.05 compared to the control; ^#^ *P* < 0.05 compared to the BCA group.

A wound-healing assay was performed to evaluate the migration of mature osteoblasts. Cells were incubated in the presence or absence of 10^−6^ M BCA for 2 days. The results presented in Figure 4(a) show that 10^−6^ M BCA enhanced cell migration. Furthermore, the BCA-induced increase ALP activity was completely blocked by the protein synthesis inhibitor, cycloheximide (10^−6^ M), suggesting that the response was dependent on de novo protein synthesis (Figure 4(b)) (*P* < 0.05). Mature osteoblasts were grown in 24-well plates for 7 days in the presence or absence of BCA and then stained with von Kossa stain to examine mineralization.  Figure 4(c) shows that the number of mineralized nodules (right panel) and the area of mineralization (left panel) were significantly increased by BCA. 

### 3.9. Osteoclast and Bone Resorption

Osteoclastogenic agent-induced osteoclasts were incubated with or without BCA for 7 days to detect the resorption pits. A remarkable and significant decrease in osteoclast resorption pits (*P* < 0.05) was observed (Figure 4(d)).

## 4. Discussion

The present study was undertaken to evaluate the effectiveness of BCA on preventing bone loss from estrogen depletion in OVX rats. Our results support previous observations pertaining to the positive effects of BCA on increasing total BMD, BMC, and BV/TV of the femur. The protective effect was manifested by the enhancement of growth and activity of osteoblasts, and repression of growth and activity of osteoclasts, and thereby reducing bone turnover. BCA at a concentration of 10^−6^ M exerted the greatest biological activity.

Bone metabolism is regulated by functions of osteoblasts and osteoclasts which are localized on bone tissues. In our previous study and this present study was shown that the isoflavone genistein and BCA molecules possess similar action in the regulation of bone metabolism [4]. In general, ER*α* is expressed in breast, uterus, and ovarian, whereas ER*β* is expressed in bone and blood vessels. Genistein can bind to ER*α* to increase uterine weight [31] and bind to ER*β* to improve bone loss [32, 33]. However, BCA is an ER*β*-selective isoflavone [15]. BCA stimulates osteoblast differentiation and mineralization, whereas osteoclast differentiation and bone resorption were suppressed (Figures 3 and 4) and no proliferative effects on the uterus were observed (Table 1) [14, 16]. Thus, the data support that BCA is a selective ER*β* modulatory-like activity. In addition, BCA binds to ER*β* with considerably less affinity than genistein [34]. This may be attributed to the presence of the 4′-methoxy group on BCA. The difference in binding affinities to ERs suggests that BCA and genistein may have different biological activities. 

Osterix, collagen type I, ALP, and osteocalcin are markers of the four stages of bone formation [35, 36] representing, respectively, the mesenchymal stem cell to preosteoblast stage, the osteoblastic proliferation stage, differentiation stage, and mineralization stage. The levels of mRNAs for all four genes were increased by BCA treatment. Biochemical measurements of bone turnover provide an objective assessment of disease activity and the response to treatment. The urinary excretion of DPD crosslinks is a marker of bone resorption. The urinary DPD/creatine ratio can be used to evaluate the bone turnover in OVX rats. Ovariectomy significantly increased urinary DPD excretion (*P* < 0.01) compared to control values. This increase was suppressed by both E2 and BCA. These results demonstrate that BCA prevents bone loss, probably as a result of decreased bone turnover. IL-1*β* and TNF-*α* are generally recognized as osteoresorptive factors [37]: they play a critical causal role in inducing bone loss [38] by stimulating osteoclastogenesis [39] and enhancing bone resorption via the induction of RANKL in osteoblasts and the induction of osteoclast maturation [40]. Increased bone remodeling with estrogen deficiency is mediated by the production of cytokines such as IL-1*β* and TNF-*α*. Therefore, the inhibitory effect of BCA on bone resorption may be associated with its anti-inflammatory effects. That is, the inhibition of IL-1*β* and TNF-*α* may lead to the inhibition of osteoclast differentiation and related activities. Moreover, the decrease in IL-1*β* and TNF-*α* reflected in the downregulation of bone cell expression of RANKL, and the upregulation of OPG expression resulted in decreased osteoclastic maturation. We found no change in serum inorganic phosphate and calcium levels in all groups, suggesting that estrogen involvement in the control of phosphate and calcium homeostasis is probably not implicated in the bone loss effects.

Bone remodeling requires a precise balance between resorption and formation. The OPG/RANK/RANKL signaling pathway is a key to regulating and maintaining the balance between the activity of osteoblasts and osteoclasts in order to prevent bone loss and to ensure normal bone turnover [41]. The mRNA levels of OPG, which is secreted by osteoblasts and is an osteoclast-activation inhibitor that acts by binding to RANKL [42], were increased by BCA. Conversely mRNA levels for TRAP, a marker of osteoclast differentiation [43], and RANKL, an osteoclast activating factor which induces NF*κ*B-p65 translocation leading to osteoclast formation [44], were decreased by BCA. The ratio of RANKL/OPG expression decreased, indicating that BCA improves bone formation, inhibits bone resorption, and decreases bone remodeling. These results show that BCA has a potent inhibitory effect at all stages of osteoclast differentiation and formation and that BCA acts on both preosteoclasts and mature osteoclasts. Furthermore, its effect appeared to be greater than that of E2. The importance of bone turnover modulation was mediated by BCA, suggesting that BCA may be a potential therapeutic drug.

Although the biochemical parameters and expression of osteogenic marker genes partly showed that BCA supplementation was more effective than E2 treatment in the present study, this might be explained by the relatively short duration of the study, or by examination of the femur tissue alone, which was not the representative of the entire physiological state. However, our results demonstrated that BCA supplementation protected against bone loss in ovariectomized rats over the time period examined in this study.

In addition, a commercial isoflavone product, BCA, offered the advantage of being relatively inexpensive, particularly compared to genistein and daidzein. We found that BCA treatment for osteoporosis was similar to that by genistein [4]; thus it may be possible to supplement with BCA tablets or isoflavone extracts manufactured from red clover.

In conclusion, BCA may provide an alternative strategy to augment bone mass, and BCA supplementation could be an alternative to HRT for the prevention of osteoporosis, especially given its range of biological effects and lack of cytotoxicity.

## Figures and Tables

**Table 1:** Effect of treatments on body weight and uterine weight change.

Treatment group (*n* = 10)	Body weight			Uterine weight
	Before treatment (g)	After treatment (g)	Increase rate (%)	(g)
Sham	290.0 ± 21.6	387.9 ± 19.6	33.6 ± 5.0^b^	0.45 ± 2.1^b^
OVX	298.0 ± 11.7	486.7 ± 18.4	63.8 ± 7.2^a^	0.27 ± 2.6^a^
OVX + E2	295.7 ± 16.2	395.7 ± 15.9	34.9 ± 4.8^b^	0.41 ± 3.4^b^
OVX + BCA	290.0 ± 13.1	380.0 ± 18.9	31.0 ± 3.4^b^	0.29 ± 2.1

Values are expressed as mean ± SD. Means within a column with different superscript letters are significantly different (*P* < 0.05, ANOVA and the Fisher test).

^a^ *P* < 0.05, when compared with the sham groups; ^b^ *P* < 0.05, when compared with the OVX group.

**Table 2:** Effect of treatments on femoral bone mineral density (BMD), bone mineral content (BMC), and BV/TV of the left femur.

Treatment group (*n* = 10)	Bone mineral density (BMD) (mg/cm^2^)	Bone mineral content (BMC) (g)	Bone volume (BV/TV) (%)
Sham	192.0 ± 8.0^b^	0.48 ± 0.4^b^	35.5 ± 5.4^b^
OVX	164.0 ± 6.7^a^	0.37 ± 0.3^a^	12.6 ± 3.3^a^
OVX + E2	185.0 ± 5.0^b^	0.44 ± 0.5^b^	28.7 ± 4.3^b^
OVX + BCA	188.0 ± 8.6^b^	0.48 ± 0.6^b^	31.8 ± 4.5^b^

BMD (mg/cm^2^) and BMC (g) were measured by Dual Energy X-Ray Absorptiometry. Values are expressed as means ± SD. Means within a column with different superscript letters are significantly different (*P* < 0.05, ANOVA and the Fisher test). ^a^ *P* < 0.05, when compared with the sham groups; ^b^ *P* < 0.05, when compared with the OVX group.

**Table 3:** Bone-related parameters of serum and urine in rats after treatment.

Treatment group (*n* = 10)	Inorganic phosphate (mg/dL)	Calcium (mg/dL)	TNF-*α* (pg/mL)	IL-1*β* (pg/mL)	DPD/creatine (nM/mM)
Sham	7.2 ± 0.8	7.4 ± 1.3	20 ± 2.1^b^	23 ± 4.9^b^	92 ± 6.1^b^
OVX	7.3 ± 1.2	7.7 ± 1.5	76 ± 4.3^a^	44 ± 5.6^a^	148 ± 8.5^a^
OVX + E2	7.0 ± 1.1	7.3 ± 1.7	10 ± 1.9^b^	18 ± 5.1^b^	82 ± 4.1^b^
OVX + BCA	7.1 ± 0.7	7.4 ± 1.2	11 ± 1.6^b^	20 ± 2.3^b^	79 ± 5.2^b^

Values are expressed as means ± SD. Differences among treatment groups were evaluated using a one-way ANOVA followed by Fisher's test. ^a^ *P* < 0.05, when compared with the Sham groups; ^b^ *P* < 0.05, when compared with the OVX group. OVX: ovariectomized.
